# Preparation of Hollow Fe_2_O_3_ Nanorods and Nanospheres by Nanoscale Kirkendall Diffusion, and Their Electrochemical Properties for Use in Lithium-Ion Batteries

**DOI:** 10.1038/srep38933

**Published:** 2016-12-13

**Authors:** Jung Sang Cho, Jin-Sung Park, Yun Chan Kang

**Affiliations:** 1 Department of Materials Science and Engineering, Korea University, Anam-Dong, Seongbuk-Gu, Seoul 136-713, Republic of Korea; 2 Department of Engineering Chemistry, Chungbuk National University, Chungbuk 361-763, Republic of Korea

## Abstract

A novel process for the preparation of aggregate-free metal oxide nanopowders with spherical (0D) and non-spherical (1D) hollow nanostructures was introduced. Carbon nanofibers embedded with iron selenide (FeSe) nanopowders with various nanostructures are prepared via the selenization of electrospun nanofibers. Ostwald ripening occurs during the selenization process, resulting in the formation of a FeSe-C composite nanofiber exhibiting a hierarchical structure. These nanofibers transform into aggregate-free hollow Fe_2_O_3_ powders via the complete oxidation of FeSe and combustion of carbon. Indeed, the zero- (0D) and one-dimensional (1D) FeSe nanocrystals transform into the hollow-structured Fe_2_O_3_ nanopowders via a nanoscale Kirkendall diffusion process, thus conserving their overall morphology. The discharge capacities for the 1000^th^ cycle of the hollow-structured Fe_2_O_3_ nanopowders obtained from the FeSe-C composite nanofibers prepared at selenization temperatures of 500, 800, and 1000 °C at a current density of 1 A g^−1^ are 932, 767, and 544 mA h g^−1^, respectively; and their capacity retentions from the second cycle are 88, 92, and 78%, respectively. The high structural stabilities of these hollow Fe_2_O_3_ nanopowders during repeated lithium insertion/desertion processes result in superior lithium-ion storage performances.

Hollow nanopowders with controlled size and morphology have wide applications in various fields, such as rechargeable batteries, hydrogen evolution reactions, gas sensors, catalysis, and drug delivery^1^,^2^,^3^,^4^,^5^,^6^,^7^. To date, zero- (0D), one- (1D), two- (2D), and three-dimensional (3D) hollow nanostructures with various compositions have been developed based on the requirements of the various applications^8^,^9^,^10^,^11^,^12^,^13^,^14^,^15^,^16^,^17^. Generally, spherical (0D) and non-spherical (1D, 2D, and 3D) hollow nanostructures are prepared by the application of removable organic and inorganic templates in liquid solution processes^13^,^14^,^15^,^16^,^17^. For example, organic polymers such as polystyrene (PS) and poly(methyl methacrylate) (PMMA), in addition to silica nanopowders have been widely used as sacrificial templates for hollow nanospheres (0D), as they can be easily removed, and their size can be easily controlled^18^,^19^,^20^.

Recently, nanoscale Kirkendall diffusion and Ostwald ripening processes, in which filled structures are transformed into hollow structures during heat treatment, have been applied in the preparation of hollow nanospheres (0D) in the absence of templates^21^,^22^,^23^,^24^,^25^. More specifically, metal nanospheres embedded within amorphous carbon and reduced graphene oxide were transformed into their corresponding oxide and selenide hollow nanospheres during the heat treatment stage of the nanoscale Kirkendall diffusion process^26^,^27^,^28^,^29^. However, controlling the metal oxide nanopowder dimensions through the oxidation of metallic nanopowders was challenging. Thus, to date, a simple and general process for the control of hollow nanostructure dimensions has not yet been developed. In addition, the preparation of aggregate-free metal oxide nanopowders via an electrospinning process has received little attention. Furthermore, the application of metal selenide nanomaterials with various morphological characteristics for the formation of hollow structured nanomaterials via the nanoscale Kirkendall diffusion process has yet to be examined in detail.

Nanostructured Fe_2_O_3_ materials including hollow spheres, core-branch arrays, nanosheets, and graphene hybrids have been extensively studied as the anode material for LIBs due to their high capacity, natural abundance, and environmental benignity^30^,^31^,^32^,^33^,^34^,^35^,^36^,^37^. Chen *et al*. synthesized aligned Fe_2_O_3_ nanotubes by a templating technique and proved their feasibility in anode materials for LIBs^36^. Lou *et al*. fabricated single-crystal Fe_2_O_3_ nanodiscs and microparticles containing porosity via a controlled H_2_C_2_O_4_ etching process^37^. Particles with higher porosity showed superior capacity retention due to effective buffering of volume changes during repetitive cycling.

Thus, we herein investigate the nanoscale Kirkendall diffusion process for the preparation of metal oxide nanopowders with hollow nanostructures of equal compositions but different dimensions (i.e., 0D and 1D). No additional surfactants or sacrificial templates were required using our procedure. Carbon nanofibers embedded with metal selenide nanopowders exhibiting nanostructures of different dimensions were prepared via selenization of the electrospun nanofibers. A subsequent oxidation process under air produced the desired aggregate-free metal oxide nanopowders via the nanoscale Kirkendall diffusion process. Finally, the morphological and electrochemical properties of the iron oxide (Fe_2_O_3_) nanopowders, which were selected as the initial target material, were systematically studied.

## Results and Discussion

**Figure 1:**
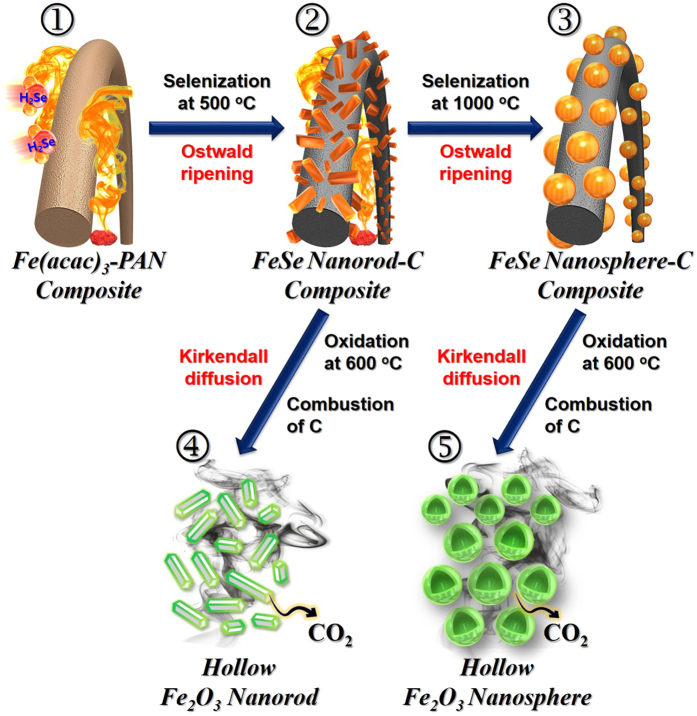
Formation mechanism of the hollow-structured Fe_2_O_3_ nanopowders with 0D and 1D structure.

**Figure 2:**
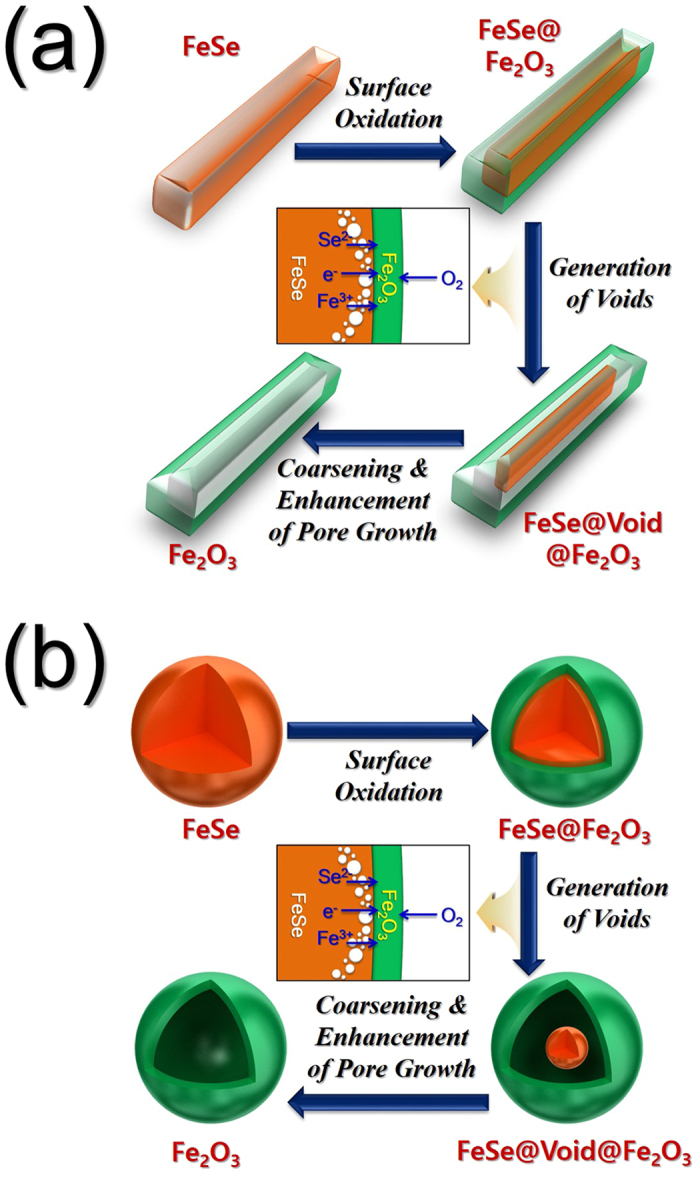
Conversion reaction of the FeSe filled structure into Fe_2_O_3_ hollow structure by nanoscale Kirkendall diffusion effect. (**a**) hollow-structured Fe_2_O_3_ nanopowder with 1D and (**b**) hollow-structured Fe_2_O_3_ nanopowder with 0D.

**Figure 3:**
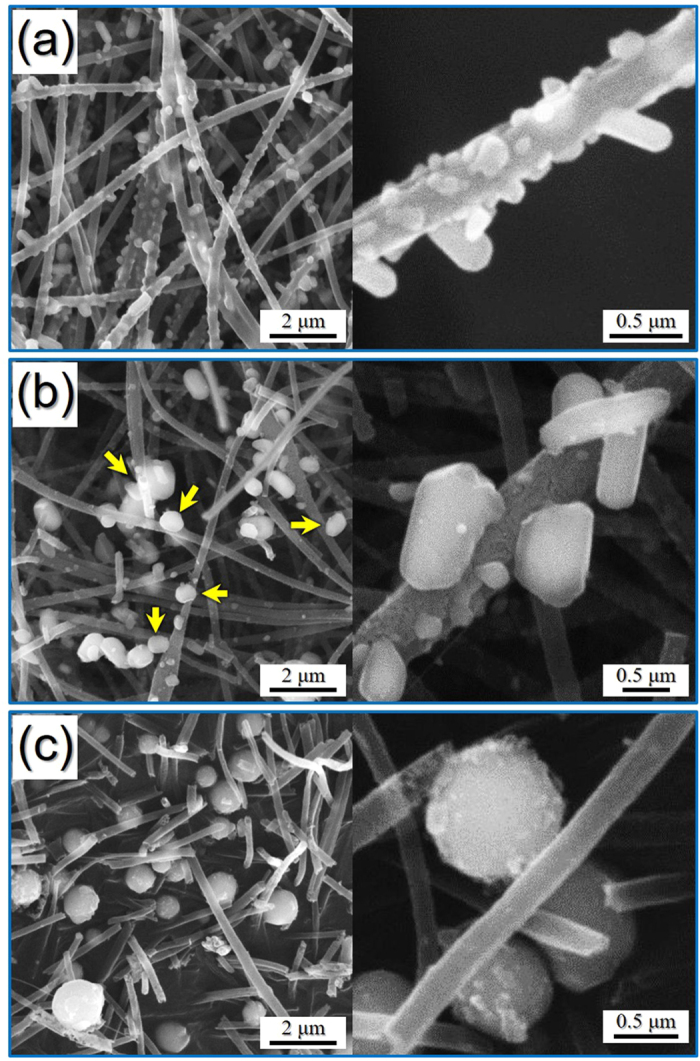
Morphologies of the FeSe-carbon composite nanofibers obtained after different selenization temperatures. (**a**) selenization at 500 °C, (**b**) selenization at 800 °C, and (**c**) selenization at 1000 °C.

**Figure 4:**
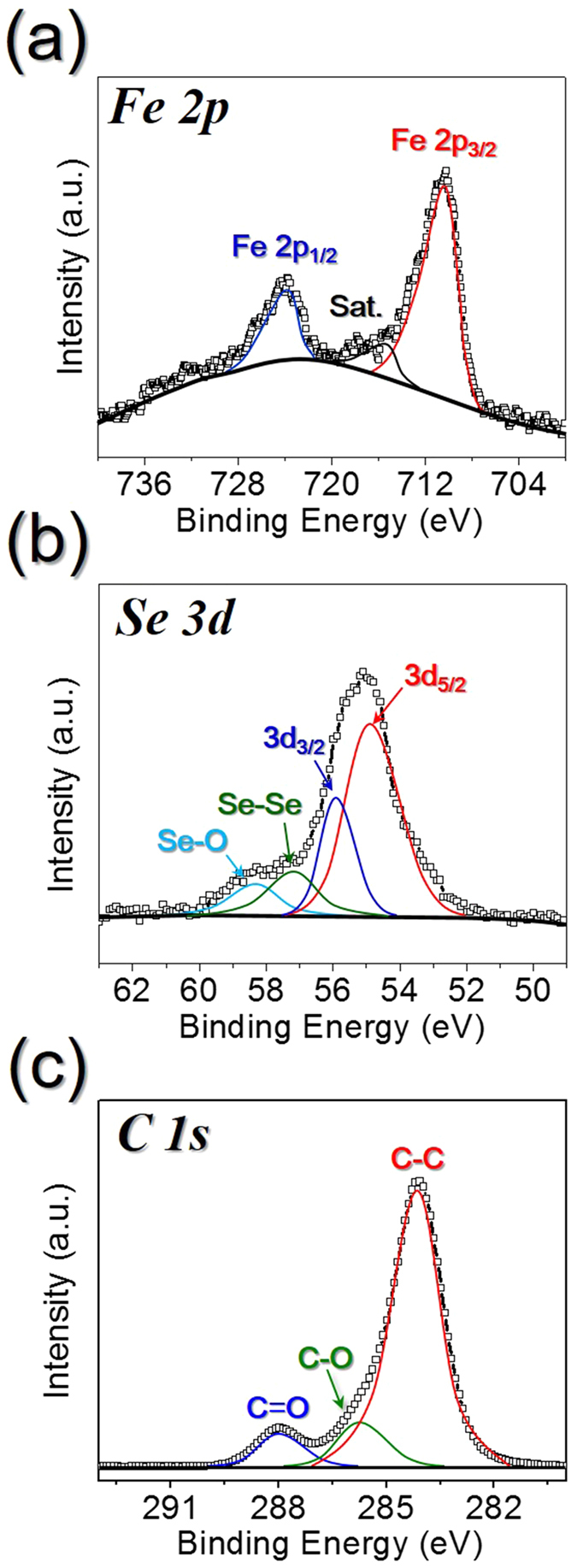
XPS spectra of the FeSe-C composite nanofibers obtained after selenization at 800 °C. (**a**) Fe 2p spectrum, (**b**) Se 3d spectrum, and (**c**) C 1 s spectrum.

**Figure 5:**
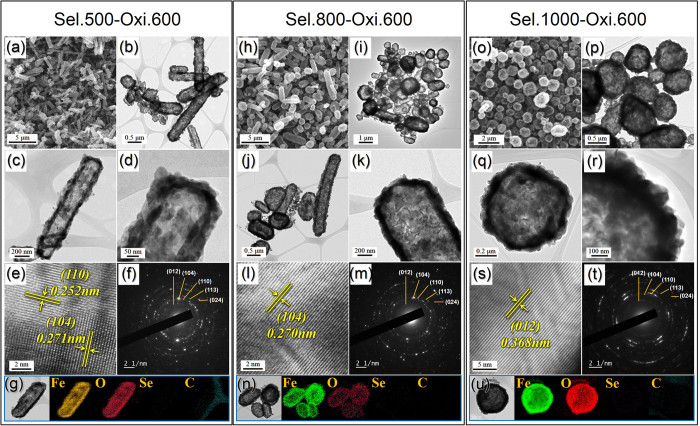
Morphologies, SAED pattern, and elemental mapping images of the hollow-structured Fe_2_O_3_ nanopowders after oxidation at 600 °C, from the FeSe-C composite nanofibers selenized at (**a–g**) 500 °C, (**h–n**) 800 °C, and (**o–u**) 1000 °C: (**a,h,o**) SEM images, (**b–d,i–k,p–r**) TEM images, (**e,l,s**) HR-TEM images, (**f,m,t**) SAED patterns, and (**g,n,u**) elemental mapping images.

Figure 1 outlines the mechanism of the formation of Fe_2_O_3_ nanopowders exhibiting hollow nanostructures of different dimensions via the nanoscale Kirkendall diffusion process. Following the selenization processes at different temperatures (i.e., 500, 800, or 1000 °C), the electrospun nanofibers (Fig. 1-①) were transformed into the hierarchical nanostructures. Selenization of the iron components located close the nanofiber surface resulted in the formation of FeSe nanocrystals during the early stages of the process. Ostwald ripening then occurred during further selenization to yield the hierarchical FeSe-C composite nanofiber. In this process, the ultrafine FeSe nanocrystals formed inside the carbon nanofiber diffused to the surface to produce FeSe crystals via Ostwald ripening. Complete selenization at 500 °C resulted in the carbon nanofiber being uniformly embedded with ultrafine FeSe nanocrystals (Fig. 1-②). However, at higher selenization temperatures (Fig. 1-③), crystal growth occurred via the segregation of nanocrystals and spheroidization due to melting. Finally, the hierarchical FeSe-C nanofibers transformed into hollow aggregate-free Fe_2_O_3_ nanopowders (Fig. 1-④ and 1-⑤) via the complete combustion of carbon and oxidation of FeSe. Furthermore, as shown in Fig. 2, FeSe nanocrystals with 0D and 1D structures transformed into the hollow-structured Fe_2_O_3_ nanopowders via a nanoscale Kirkendall diffusion process, thus conserving their overall morphology. For simplicity, the hollow Fe_2_O_3_ nanopowders obtained from the FeSe-C composite nanofibers prepared at 500, 800, and 1000 °C are referred to as “Sel.500-Oxi.600,” “Sel.800-Oxi.600,” and “Sel.1000-Oxi.600,” respectively.

The mechanism of formation of the hollow Fe_2_O_3_ nanopowders with 1D (Fig. 2a) and 0D structures (Fig. 2b) was investigated based on the morphologies and crystal structures of the materials obtained in each step. The morphologies and crystal structures of the electrospun nanofibers stabilized at 120 °C under air are shown in Figure S1. The nanofibers exhibited a clean surface structure and an amorphous-like crystal structure with small Fe_3_O_4_ crystalline peaks, and transformed into the desired hierarchical structured nanofibers following selenization at 500, 800, and 1000 °C, as shown in Fig. 3. During this selenization process, the carbonization of PAN resulted in the formation of carbon nanofibers. As confirmed by XRD studies (Figure S2), the nanocrystals decorated on the carbon nanofiber surfaces were iron selenide (FeSe). Interestingly, although the composite nanofibers obtained at a selenization temperature of 500 °C exhibited a pure hexagonal FeSe phase, those obtained at 800 and 1000 °C exhibited mixed hexagonal FeSe and tetragonal FeSe crystal structure phases. As outlined previously (Fig. 1), FeSe nanocrystals formed over the carbon nanofibers during the Ostwald ripening process. Indeed, following selenization at 500 °C, rod-like FeSe nanocrystals were obtained, as shown in the SEM images of the FeSe-C composite nanofibers provided in Fig. 3a. However, at higher temperatures (i.e., 800 °C), a number of FeSe nanorods underwent spheroidization, as indicated by the arrows in Fig. 3b. Furthermore, upon increasing the selenization temperature to 1000 °C, complete spheroidization took place, resulting in the carbon nanofibers being decorated by spherical nanocrystals, as shown in Fig. 3c. Moreover, due to Ostwald ripening, upon increasing the selenization temperature, the size of the FeSe nanocrystals increased due to crystal growth. However, even at high temperatures, the carbon nanofibers minimized crystal growth, giving a mean size of 0.9 μm for the FeSe nanospheres, as determined from the SEM images.

XPS was then employed to determine the chemical states and molecular environments of the FeSe-C composite obtained at 800 °C, as shown in Fig. 4. In the Fe 2p spectrum shown in Fig. 4a, the main peaks were observed at the characteristic FeSe binding energies of 710.2 eV (Fe 2p_3/2_) and 724.1 eV (Fe 2p_1/2_)^38^,^39^,^40^. In addition, in the Se 3d spectrum shown in Fig. 4b, signals were observed at binding energies of 54.5 and 55.3 eV, corresponding to Se 3d_5/2_ and Se 3d_3/2_, respectively. Signals corresponding to the oxidized-Se (Se-O) and metalloid Se (Se-Se) bonds were also observed in the XPS spectrum of Se, with the former due to the partial oxidation of metalloid Se under atmospheric conditions, and the latter likely due to the deposition of small amounts of metalloid Se within the porous carbon nanofiber during the selenization process^39^. Furthermore, in the C 1 s XPS spectrum (Fig. 4c), peaks corresponding to sp_2_-bonded carbon (C–C), epoxy and alkoxy groups (C–O), and carbonyl and carboxylic (C = O) components were observed at 284.2, 286.5, and 288.0 eV, respectively. As the peak corresponding to the C–C bond exhibited the highest intensity, and those corresponding to the C–O and C = O bonds exhibited particularly low intensities, this indicates that graphitic carbon was formed during the selenization process^40^.

Following oxidation at 600 °C, the FeSe-C composite nanofibers formed at different selenization temperatures transformed into pure Fe_2_O_3_ nanopowders with various morphologies. The XRD patterns shown in Figure S3 confirm the complete oxidation of FeSe into Fe_2_O_3_ for all FeSe nanocrystal morphologies during the oxidation process. In addition, the SEM and TEM images shown in Fig. 5a–g reveal the formation of aggregate-free hollow Fe_2_O_3_ nanorods via the complete oxidation of the FeSe-C composite nanofibers that were obtained at a selenization temperature of 500 °C. From the SEM image, the Fe_2_O_3_ nanorods measured ~0.49 μm (mean width) by 3.40 μm (mean length), while the mean thickness of the hollow shell was determined to be 63 nm from the TEM images. The high-resolution TEM image shown in Fig. 5e shows clear lattice fringes separated by 0.25 and 0.27 nm, which correspond to the (110) and (104) crystal planes of rhombohedral hematite Fe_2_O_3_, respectively. The morphologies of the Sel.800-Oxi.600 and Sel.1000-Oxi.600 Fe_2_O_3_ powders are shown in Fig. 5h–n and o–u, respectively. Indeed, the SEM and TEM images shown in Fig. 5h–k of the Sel.800-Oxi.600 revealed two types of Fe_2_O_3_ hollow nanopowders, based on spherical and rod-like shapes, while the hollow Fe_2_O_3_ nanopowders shown in Fig. 5o–r of the Sel.1000-Oxi.600 exhibited only a spherical shape. In addition, the high-resolution TEM images shown in Fig. 5i and s show clear lattice fringes separated by 0.37 and 0.27 nm, which correspond to the (012) and (104) crystal planes of Fe_2_O_3_, respectively. The FeSe crystals obtained from the FeSe-C composite nanofibers that were transformed into hollow Fe_2_O_3_ powders by the nanoscale Kirkendall diffusion process are outlined in Fig. 2. Surface oxidation of the FeSe crystal resulted in the formation of an FeSe@Fe_2_O_3_ powder, which exhibited a core-shell structure. The outward diffusion of the smaller Fe cations (Fe^2+^ = 76 pm, Fe^3+^ = 65 pm) and Se anions (184 pm) occurred more rapidly than the inward diffusion of O_2_ gas. Thus, the formation of an SeO_2_ layer over the hollow powder due to the oxidation of diffused Se was eliminated by volatilization at an oxidation temperature of 600 °C. Therefore, complete diffusion of the Fe and Se components to the outside surface of the powder resulted in the formation of hollow Fe_2_O_3_ powders. The selected area electron diffraction (SAED) pattern and elemental mapping images shown in Fig. 5 therefore confirm the formation of single phase Fe_2_O_3_ powders containing no Se or C impurities.

**Figure 6:**
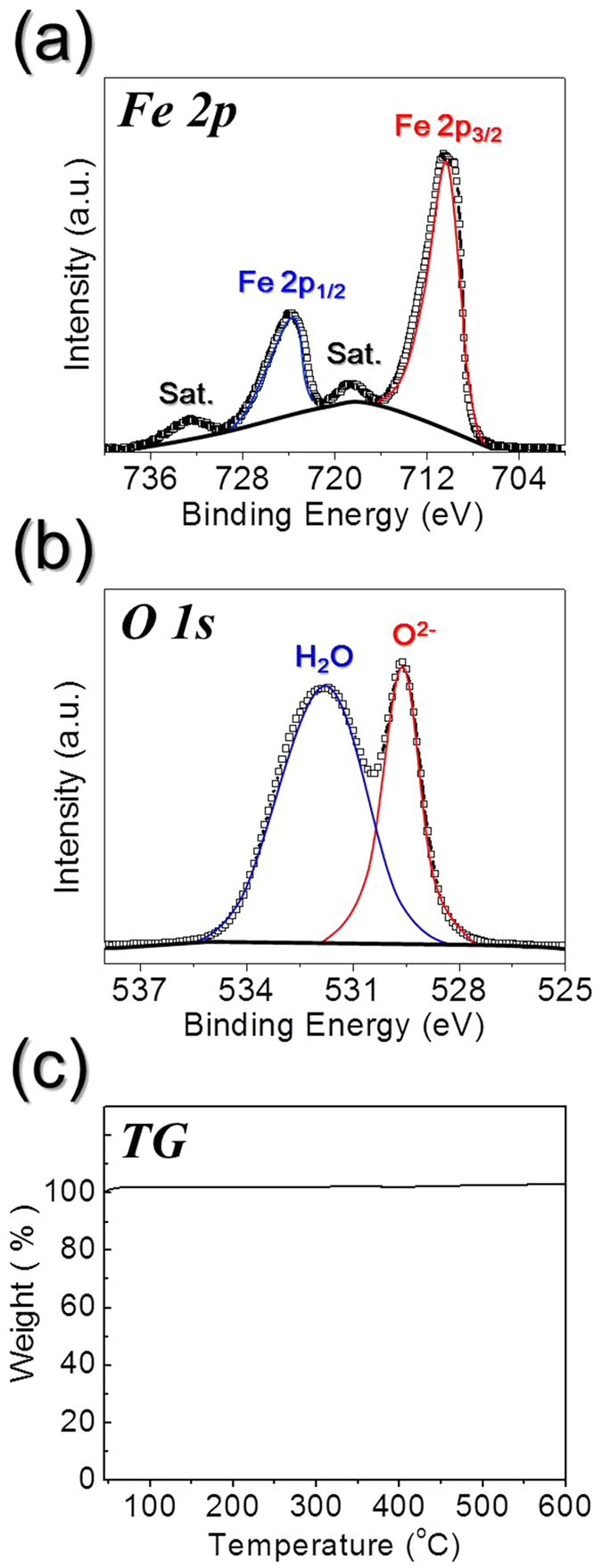
XPS spectra and TG curve of the hollow-structured Fe_2_O_3_ nanopowders after oxidation at 600 °C from the FeSe-C nanofibers selenized at 800 °C. (**a**) XPS Fe 2p spectrum, (**b**) XPS O 1 s spectrum, and (**c**) TG curve.

The XPS spectra and TG curve of the hollow-structured Sel.800-Oxi.600 nanopowders are shown in Fig. 6. In the Fe 2p spectrum shown in Fig. 6a, the major signals are observed at binding energies of 711.2 eV (Fe 2p_3/2_) and 724.2 eV (Fe 2p_1/2_), which are characteristic of Fe_2_O_3_^41^,^42^,^43^,^44^,^45^. In addition, the O 1 s spectrum shown in Fig. 6b exhibits two peaks centered at 529.7 eV and 531.6 eV, which could be assigned to the oxygen atoms in the Fe_2_O_3_ lattice and to adsorbed water present on the powder surfaces^45^. Thus, combined with the observation of Fe 2p and O 1 s signals as discussed above, these results confirmed the formation of pure Fe_2_O_3_ phase. No components related to zero-valent Fe or Fe^2+^ were observed. Furthermore, the TG curve shown in Fig. 6c confirmed no distinct weight loss, with an increase in mass being observed at temperatures <600 °C, revealing the phase purity of the obtained Fe_2_O_3_ powders. Moreover, as shown in Figure S4, the BET surface areas of the hollow Sel.500-Oxi.600, Sel.800-Oxi.600, and Sel.1000-Oxi.600 nanopowders were 66, 61, and 27 m^2^ g^−1^, respectively.

**Figure 7:**
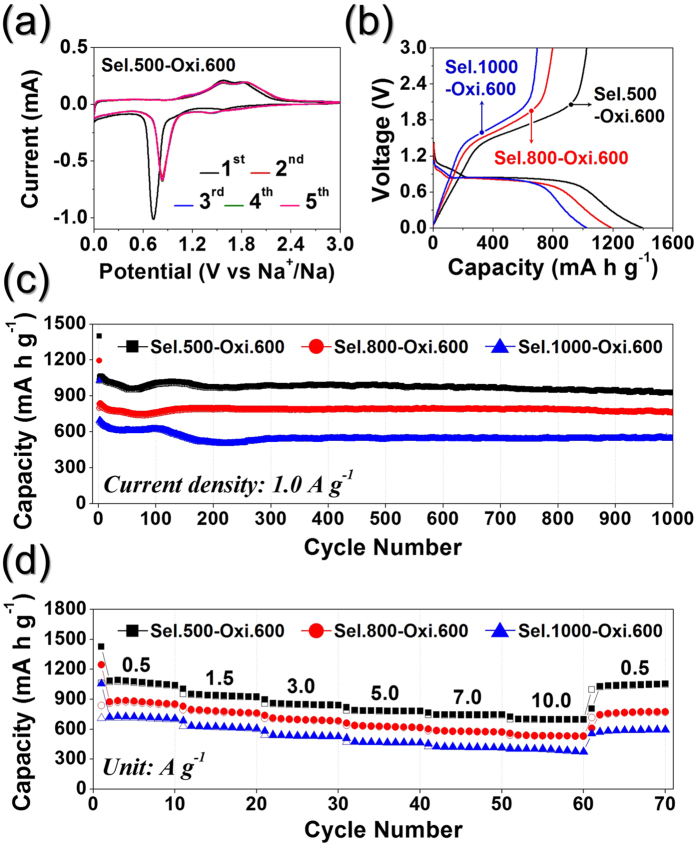
Electrochemical properties of the hollow-structured Fe_2_O_3_ nanopowders. (**a**) CV curves of the Sel.500-Oxi.600, (**b**) first discharge-charge profiles at a current density of 1.0 A g^−1^, (**c**) cycling performances at a current density of 1.0 A g^−1^, and (**d**) rate performances.

The electrochemical properties for lithium-ion storage of the hollow 0D and 1D Fe_2_O_3_ nanopowders are shown in Fig. 7. The cyclic voltammetry (CV) curves of the hollow Fe_2_O_3_ powders for the first 5 cycles at a scan rate of 0.07 mV s^−1^ over the potential range of 0.001–3 V are shown in Figs 7a and S5. The first sharp reduction peak observed at ~0.7 V in the initial cathodic sweep corresponded to the conversion of Fe_2_O_3_ to metallic Fe and Li_2_O^46^,^47^,^48^. The two broad oxidation peaks at ~1.6 and 1.8 V in the anodic sweeps were attributed to the oxidation of Fe^0^ to Fe^2+^ and Fe^2+^ to Fe^3+^, respectively^47^,^48^. After the first cycle at a current density of 1.0 A g^−1^, the sharp reduction peak moved to ~0.8 V due to the conversion of Fe_2_O_3_ into ultrafine nanocrystals during the initial cycle^48^,^49^,^50^. In addition, Fig. 7b shows the initial charge and discharge profiles of the hollow 0D and 1D Fe_2_O_3_ nanopowders at a current density of 1 A g^−1^. The three samples yielded similar initial charge/discharge profiles irrespective of their morphologies. A plateau at ~0.84 V due to conversion of Fe_2_O_3_ to metallic Fe and Li_2_O was observed in the initial discharge process of all three samples. In addition, the initial discharge capacities of the hollow Sel.500-Oxi.600, Sel.800-Oxi.600, and Sel.1000-Oxi.600 nanopowders were 1399, 1194, and 1028 mA h g^−1^, respectively, their initial charge capacities were 1025, 796, and 694 mA h g^−1^, respectively, and their corresponding Coulombic efficiencies were 73, 67, and 68%, respectively. Indeed, the hollow Fe_2_O_3_ nanopowders with 1D structure formed by oxidation of the FeSe-C composite nanofibers obtained at a selenization temperature of 500 °C exhibited the highest initial discharge capacity and Coulombic efficiency of the three samples. However, as shown in Fig. 7c, the hollow Fe_2_O_3_ nanopowders exhibited excellent cycling performances at a current density of 1 A g^−1^ irrespective of morphology. Furthermore, the discharge capacities for the 1000^th^ cycle of the hollow Sel.500-Oxi.600, Sel.800-Oxi.600, and Sel.1000-Oxi.600 nanopowders were 932, 767, and 544 mA h g^−1^, respectively, and their capacity retentions measured from the second cycle were 88, 92, and 78%, respectively. As shown in Fig. 7d, all three hollow Fe_2_O_3_ powders also exhibited excellent rate performances. The final discharge capacities of the hollow Sel.500-Oxi.600 powders at current densities of 0.5, 1.5, 3.0, 5.0, 7.0, and 10.0 A g^−1^ were 1041, 926, 844, 785, 747, and 700 mA h g^−1^, respectively. Indeed, all three samples prepared at different selenization temperatures exhibited good capacity recovery when the current density returned to 0.5 A g^−1^, even after cycling at high current densities. As shown in Figure S6, the rate capability of the hollow Sel.500-Oxi.600 powders at extremely high current densities was also analyzed. The hollow 1D Fe_2_O_3_ nanopowders had final discharge capacities of 1083, 895, 801, 691, 613, 547, and 500 mA h g^−1^ at current densities of 0.5, 5.0, 10.0, 20.0, 30.0, 40.0 and 50.0 A g^−1^, respectively. In general, electrochemical properties of anode materials in LIBs are strongly affected by many factors, including their morphologies, crystal structures, crystallite sizes, and BET surface areas, etcetera. Therefore, the characteristics of the samples are summarized in Table S1. The rod-like Sel.500-Oxi.600 nanopowders showed the smallest crystallite and particle size, and the largest BET surface area among the samples. These features provide short transport lengths for Li ions and electrons, increased contact areas between the electrolyte and electrode for Li ions insertion–desertion, and efficient electron transport along the interconnected nanoparticle network, hence enhancing electrochemical properties. Consequently, Sel.500-Oxi.600 nanopowders with the smaller crystallite and particle size, and larger BET surface area exhibited enhaced the initial discharge capacity and cycling performance.

**Figure 8:**
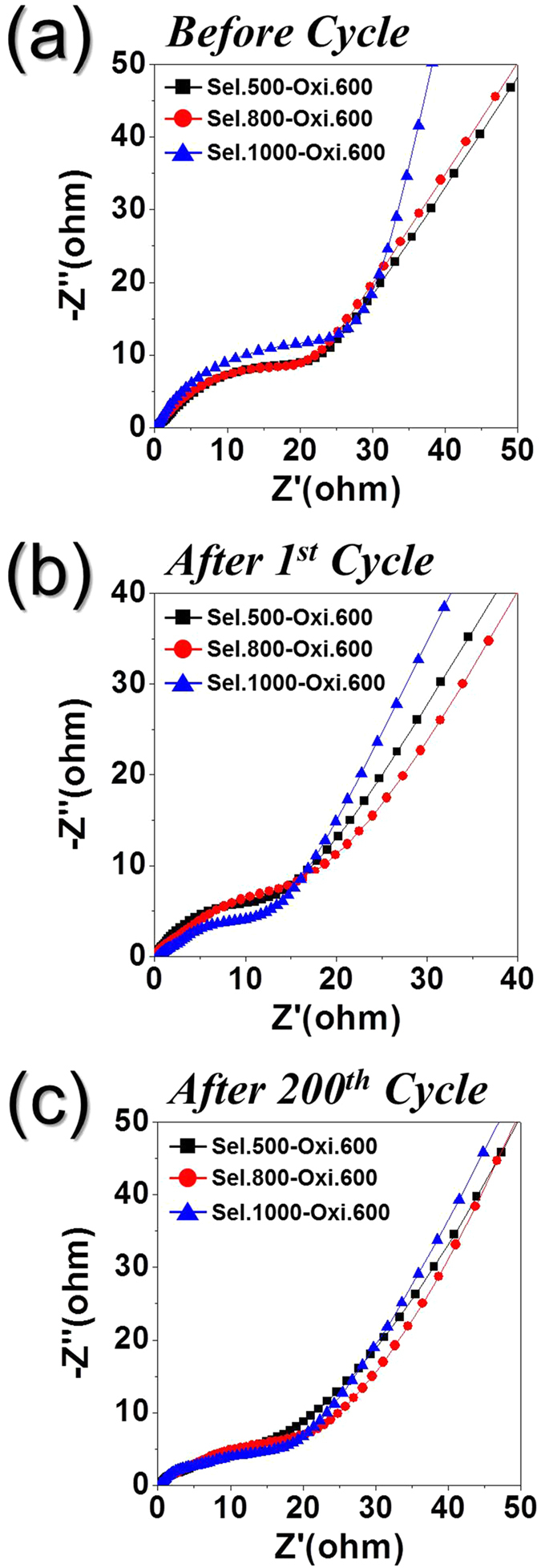
Nyquist impedance plots of the hollow-structured Fe_2_O_3_ nanopowders before and after cycling. (**a**) before cycle, (**b**) after 1^st^ cycle, and (**c**) after 200^th^ cycle.

**Figure 9:**
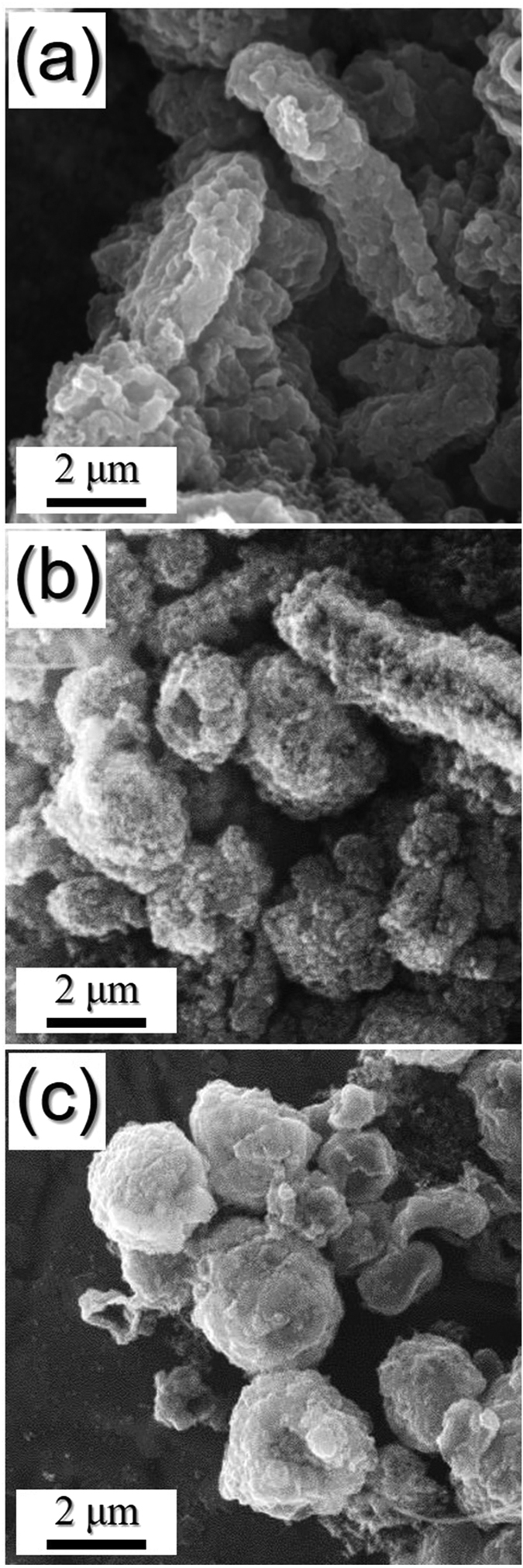
Morphologies of the hollow Fe_2_O_3_ powders obtained after 200 cycles at a current density of 1.0 A g^−1^. (**a**) Sel.500-Oxi.600, (**b**) Sel.800-Oxi.600, and (**c**) Sel.1000-Oxi.600.

The excellent Li-ion storage performances of the hollow Fe_2_O_3_ nanopowders formed from the FeSe-C composite nanofibers were confirmed by electrochemical impedance spectroscopy(EIS) measurements before and after 1 and 200 cycles. The Nyquist plots (Fig. 8) exhibit compressed semicircles in the medium-frequency range, which describe the charge-transfer resistance (*R*_*ct*_) of the electrode^51^,^52^,^53^. In addition, the hollow Sel.1000-Oxi.600 nanopowders exhibited a slightly higher charge transfer resistance than the other two samples. However, the three samples had similar charge transfer resistances after 1 and 200 cycles, with the low charge-transfer resistances of the three samples observed following the 1^st^ cycle (due to the formation of ultrafine nanocrystals) increasing slightly after 200 cycles. This is likely due to the partial structural destruction of the hollow Fe_2_O_3_ nanopowders during repeated lithium insertion/desertion, which increases the charge transfer resistances for all morphologies. Finally, as shown in Fig. 9, the overall morphologies of the hollow Fe_2_O_3_ nanopowders were maintained even after 200 cycles. The electrochemical properties of the hollow Fe_2_O_3_ nanopowders prepared from the FeSe-C composite nanofibers at different selenization temperatures were compared with those of hollow Fe_2_O_3_ materials reported in the literatures, and the results were summarized in Table S2. The hollow Fe_2_O_3_ nanopowders prepared in this study showed superior lithium-ion storage performances compared to those of the other Fe_2_O_3_ materials prepared from various processes even at a high current density of 1.0 A g^−1^ during 1000 cycles.

## Conclusions

A new process for the preparation of aggregate-free iron oxide nanopowders with spherical (0D) and non-spherical (1D) hollow nanostructures was investigated and developed. Ostwald ripening and the nanoscale Kirkendall diffusion process were successfully applied to the electrospinning process to synthesize the desired hollow nanopowders. The FeSe-C composite nanofibers formed via Ostwald ripening were transformed into hollow Fe_2_O_3_ nanopowders with either 0D or 1D nanostructures via a one-pot oxidation process through nanoscale Kirkendall diffusion, with selenization temperature of 500, 800, and 1000 °C being investigated. The prepared hollow Fe_2_O_3_ nanopowders exhibited excellent lithium-ion storage capabilities. We propose that the three-step process developed in this study can be efficiently applied in the preparation of other hollow metal oxide nanopowders with nanostructures of various dimensions (e.g., 0–3D) for applications such as lithium-ion batteries.

## Materials and Methods

### Sample preparation

Spherical (0D) and non-spherical (1D) Fe_2_O_3_ hollow nanostructures were prepared in three steps, namely the formation of electrospun precursor nanofibers and two subsequent post-treatment steps. Initially, Fe(acac)_3_-polyacrylonitrile (PAN) [Fe(acac)_3_–PAN] composite nanofibers were prepared as the precursor nanofibers via electrospinning. The electrospinning precursor solution was prepared by dissolving Fe(acac)_3_ (6.0 g, STREM Chemicals, 99%) and PAN (4.0 g, Sigma-Aldrich, M_w_: 150,000) in N,N-dimethylformamide (60 mL, DMF, Sigma-Aldrich, 99%) with vigorous stirring overnight. The prepared solution was loaded into a plastic syringe equipped with a 25-gauge stainless steel nozzle at a flow rate of 2 mL h^−1^. The solution was subsequently ejected and electrospun onto a drum collector covered with aluminum foil. During the electrospinning process, the distance between the tip and the collector was maintained at 20 cm, while the rotation of the drum was maintained at 100 rpm. The applied voltage between the collector and the syringe tip was 25 kV. The resulting Fe(acac)_3_–PAN composite nanofibers were stabilized at 120 °C under air for 5 h. Subsequently, the initial post-treatment step to achieve selenization of the electrospun nanofibers was conducted at 500, 800, or 1000 °C for 6 h under H_2_Se gas. H_2_Se was generated from commercial Se metal powder and H_2_ gas. In the selenization process, the Fe(acac)_3_–PAN composite nanofibers and Se metal powder were loaded into a covered alumina boat and placed in a quartz tube reactor. Selenization resulted in the formation of FeSe-carbon composite nanofibers. The subsequent oxidation process was conducted under air at 600 °C for 3 h. For simplicity, the hollow Fe_2_O_3_ powders obtained from the FeSe-C composite nanofibers prepared at 500, 800, and 1000 °C are referred to as “Sel.500-Oxi.600,” “Sel.800-Oxi.600,” and “Sel.1000-Oxi.600,” respectively.

### Characterization

The hollow Fe_2_O_3_ powder microstructures were observed by field emission scanning electron microscopy (FE-SEM, S-4800, Hitachi) and field emission transmission electron microscopy (FE-TEM, JEM-2100F, JEOL). In addition, their crystal phases were evaluated by X-ray diffractometry (XRD, X’Pert PRO MPD, PANalytical) using Cu Kα radiation (λ = 1.5418 Å) at the Korea Basic Science Institute (Daegu). X-ray photoelectron spectroscopy (XPS, Thermo Scientific K-Alpha^TM^) with a focused monochromatic Al Kα beam at 12 kV and 20 mA was used to analyze the composition of the specimens. The surface areas of the nanofibers were measured using the Brunauer–Emmett–Teller (BET) method, using N_2_ as the adsorbate gas. Thermogravimetric analysis (TGA) was performed using a Pyris 1 TGA (Perkin Elmer) at 25–650 °C with a heating rate of 10 °C min^−1^ under air.

### Electrochemical measurements

The electrochemical properties of the hollow Fe_2_O_3_ powders were analyzed by constructing a 2032-type coin cell. The anode was prepared by mixing the active material, carbon black, and sodium carboxymethyl cellulose (CMC) in a weight ratio of 7:2:1. Li metal and microporous polypropylene film were used as the counter electrode and the separator, respectively. The electrolyte was composed of 1 M LiPF_6_ dissolved in a mixture of fluoroethylene carbonate/dimethyl carbonate (FEC/DMC; 1:1 v/v). The discharge/charge characteristics of the samples were investigated by cycling over a potential range of 0.001–3 V at various current densities. Cyclic voltammograms were measured at a scan rate of 0.07 mV s^−1^. The Fe_2_O_3_-containing negative electrode measured 1.5 cm × 1.5 cm, and the mass loading of the active materials was kept approximately 1.5 mg cm^−2^ in every electrochemical test. Electrochemical impedance spectra were obtained by performing alternating current electrochemical impedance spectroscopy (EIS, ZIVE SP1) over a frequency range of 0.01 Hz to 100 kHz.

## Additional Information

**How to cite this article**: Cho, J. S. *et al*. Preparation of Hollow Fe_2_O_3_ Nanorods and Nanospheres by Nanoscale Kirkendall Diffusion, and Their Electrochemical Properties for Use in Lithium-Ion Batteries. *Sci. Rep.* **6**, 38933; doi: 10.1038/srep38933 (2016).

**Publisher's note:** Springer Nature remains neutral with regard to jurisdictional claims in published maps and institutional affiliations.

## Supplementary Material

Supporting Information
