# Supplementary material for: Preparation of Hollow Fe2O3 Nanorods and Nanospheres by Nanoscale Kirkendall Diffusion, and Their Electrochemical Properties for Use in Lithium-Ion Batteries
Source: Sci Rep. 2016 Dec 13;6:38933. doi: 10.1038/srep38933 (PMC5153625; doi:10.1038/srep38933)
Supplement: Supporting Information [file srep38933-s1.pdf]

Supporting Information

Preparation of Hollow Fe_2O_3 Nanorods and Nanospheres by Nanoscale Kirkendall Diffusion, and their Electrochemical Properties for use in Lithium-ion Batteries

Jung Sang Cho[1,2,], Jin-Sung Park[1,*], and Yun Chan Kang[1]*

J. S. Cho, J. -S. Park, and Prof. Y. C. Kang

[1] Department of Materials Science and Engineering, Korea University, Anam-Dong, Seongbuk-Gu, Seoul 136-713, Republic of Korea.

[2] Department of Engineering Chemistry, Chungbuk National University, Chungbuk 361-763, Republic of Korea

*These authors contributed equally to this work. Correspondence and requests for materials should be addressed to Y. C. Kang. (email: yckang@korea.ac.kr)

Keywords: Kirkendall diffusion, Hollow nanosphere, Hollow nanorod, Iron oxide, Lithium-ion battery

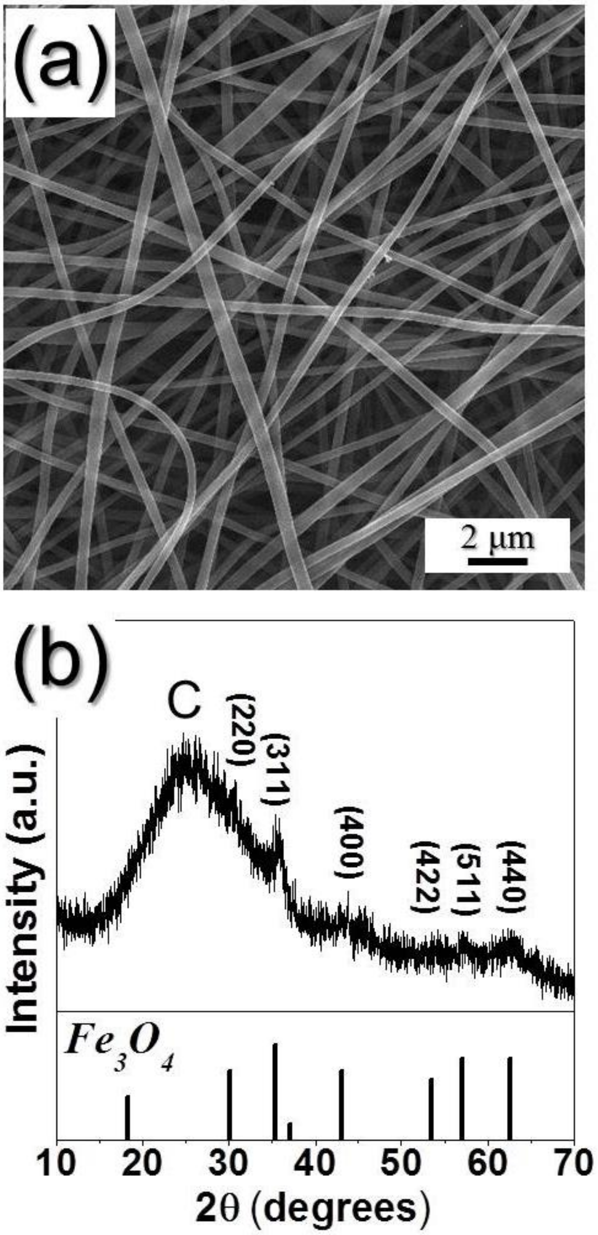

Figure S1 (a) SEM image and (b) XRD pattern of the electrospun nanofibers after strabilization at 120 ^{o}C in air.

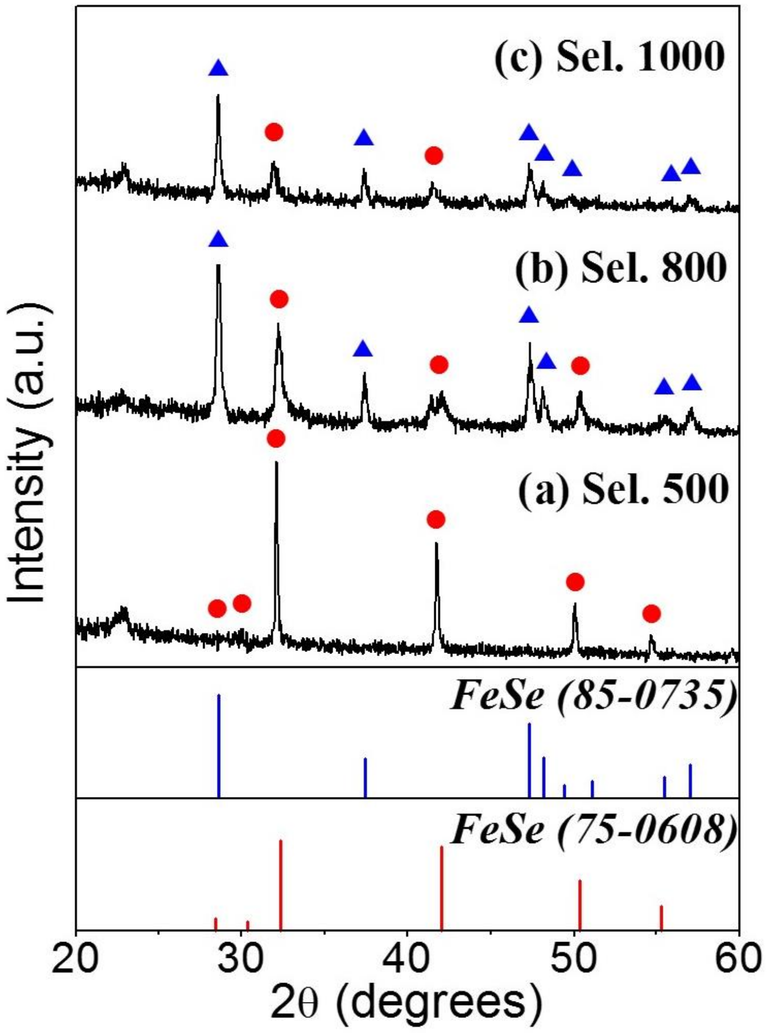

Figure S2 XRD patterns of FeSe-carbon composite nanofibers obtained at different selenization temperatures: (a) selenization at 500 ^{o}C, (b) selenization at 800 ^{o}C, and (c) selenization at 1000 ^{o}C.

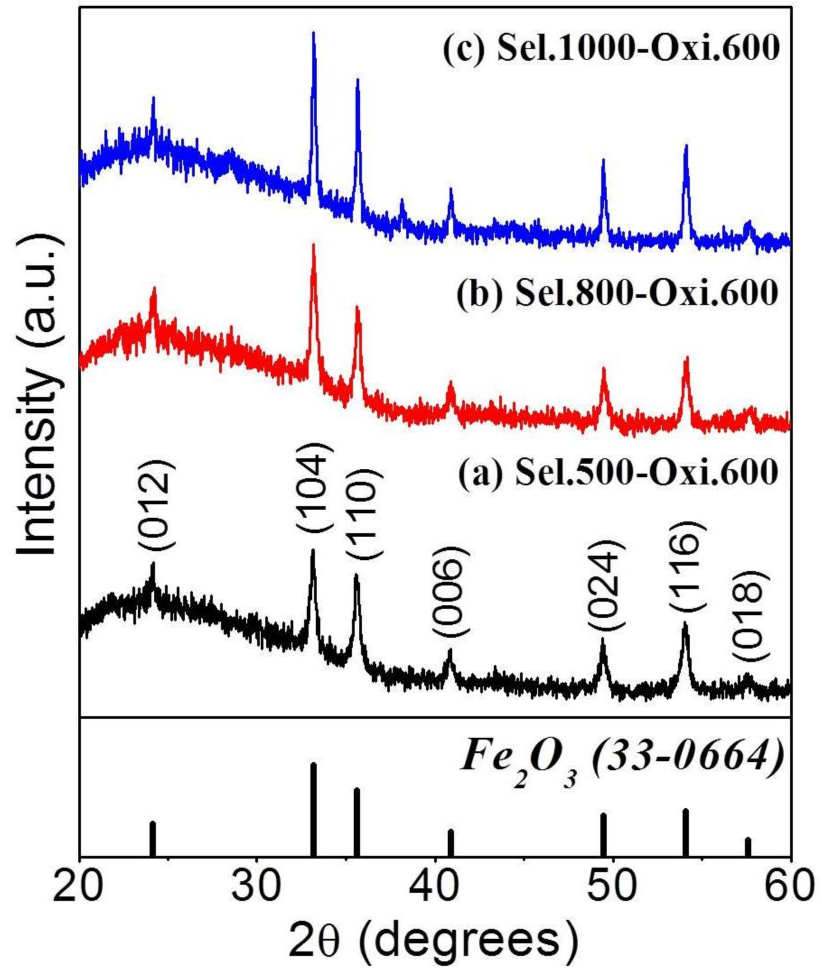

Figure S3 XRD patterns of the hollow-structured Fe_2O_3 nanopowders obtained after oxidation at 600 ^oC from the FeSe-C composite nanofibers selenized at different temperatures: (a) Sel.500-Oxi.600, (b) Sel.800-Oxi.600, and (c) Sel.1000-Oxi.600.

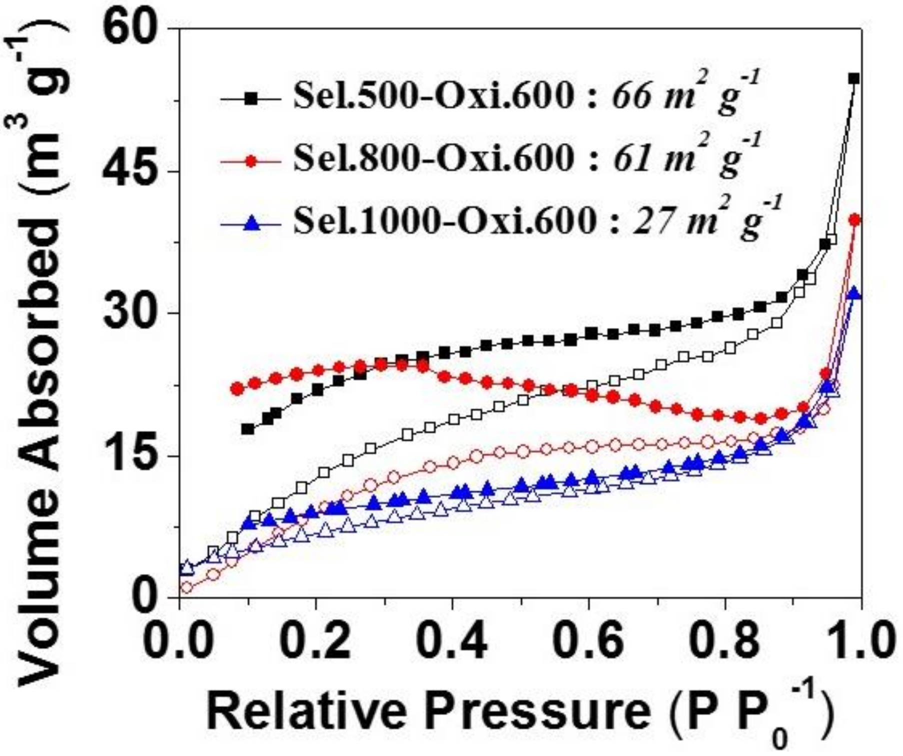

Figure S4 N_2 gas adsorption and desorption isotherms of the hollow structured Fe_2O_3 nanopowders obtained after oxidation at 600 °C from the FeSe-C composite nanofibers selenized at different temperatures.

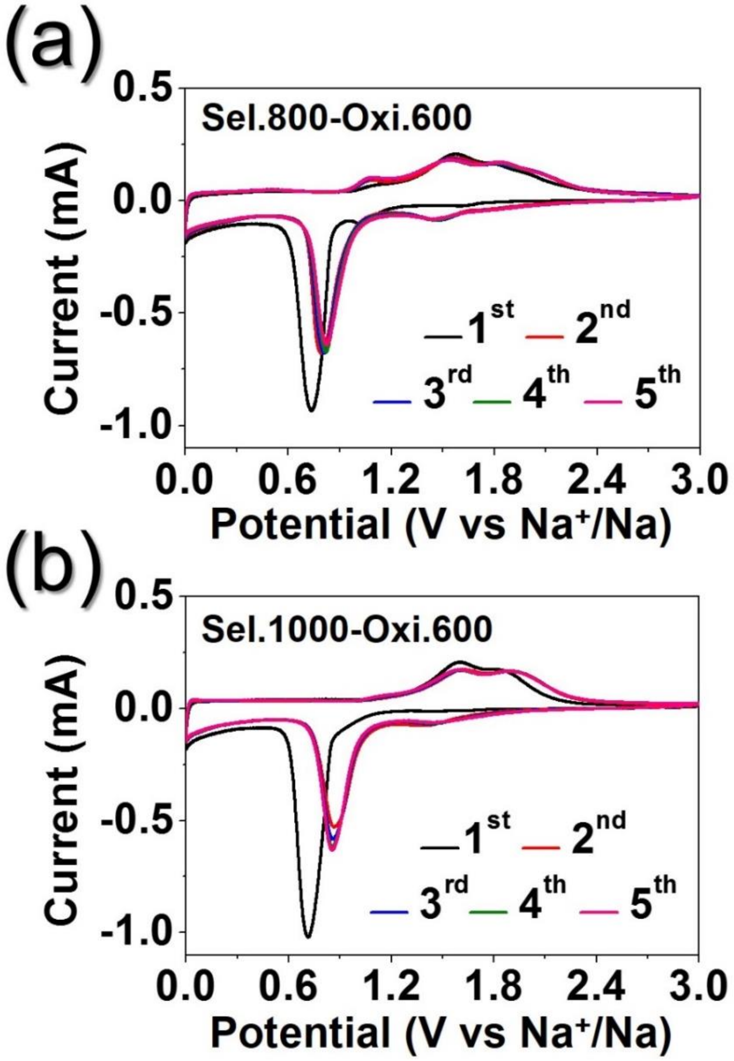

Figure S5 CV curves of the hollow-structured Fe_2O_3 nanopowders: (a) Sel.800-Oxi.600 and (b) Sel.1000-Oxi.600.

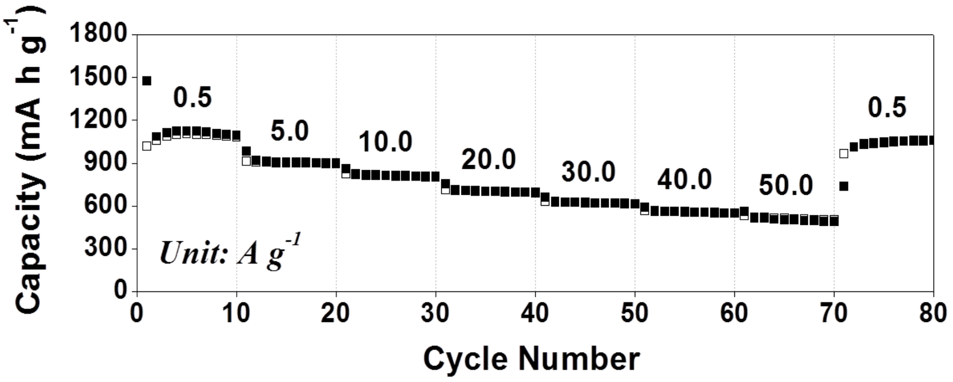

Figure S6 Rate capability of the hollow Sel.500-Oxi.600 nanopowders at extremely high current densities.

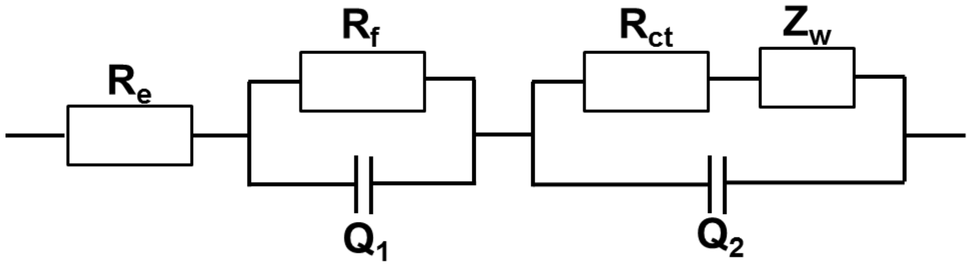

R_e : the electrolyte resistance, corresponding to the intercept of high frequency semicircle at Z_{re} axis

R_f : the SEI layer resistance corresponding to the high-frequency semicircle

Q_1 : the dielectric relaxation capacitance corresponding to the high-frequency semicircle

R_{ct}: the denote the charger transfer resistance related to the middle-frequency semicircle

Q_2 : the associated double-layer capacitance related to the middle-frequency semicircle

Z_w : the Li-ion diffusion resistance

Figure S7 Randle-type equivalent circuit model used for AC impedance fitting.

Table S1. Comparison of the features among the hollow-structured Fe_2O_3 nanopowders.

	Sel.500-Oxi.600	Sel.800-Oxi.600	Sel.1000-Oxi.600
Morphology	Hollow Nanorods	Hollow Nanorods + Hollow Nanospheres	Hollow Nanospheres
Particle Size	Rod: 0.42 x 2.91 μm	Rod: 0.92 x 3.03 μm<br>Sphere: 1.1 μm	Sphere: 0.84 μm
Crystallite Size	22.8 nm	23.2 nm	33.8 nm
BET Surface Area	66 $m^2\ g^{-1}$	61 $m^2\ g^{-1}$	27 $m^2\ g^{-1}$

Table S2 Electrochemical properties of the hollow-structured Fe_2O_3 materials as anode materials for LIBs.

Morphology	Voltage range [V]	Current rate	Initial discharge capacity [mA h g^{-1}]	Initial Coulombic efficiency [%]	Last discharge capacity [mA h g^{-1}]	Cycle number	Ref.
**Hollow nanopowders (Sel.500-Oxi.600)**	**0.001–3.0**	**1000 mA g^{-1}**	**1399**	**73**	**932**	**1,000**	**This study**
**Hollow nanopowders (Sel.800-Oxi.600)**	**0.001–3.0**	**1000 mA g^{-1}**	**1194**	**67**	**767**	**1,000**	**This study**
**Hollow nanopowders (Sel.1000-Oxi.600)**	**0.001–3.0**	**1000 mA g^{-1}**	**1028**	**68**	**544**	**1,000**	**This study**
Micron-sized spherical aggregate composed of hollow nanospheres	0.001–3.0	3000 mA g^{-1}	1267	77	854	100	S1
Hollow sphere	0.05-3.0	200 mA g^{-1}	1219	72	710	100	S2
Hollow nanoparticle	0.01-3.5	0.2 mA cm^{-2}	1186	76	700	60	S3
Hierarchical hollow sphere	0.01–3.0	500 mA g^{-1}	1255	67	815	200	S4
Microbox with Hierarchical Shell	0.01–3.0	200 mA g^{-1}	1180	71	945	30	S5
Hollow nanosphere	0.005–3.0	0.25 C	1435	69.5	690	50	S6
Hollow sphere with carbon coating	0.01–3.0	0.3 C	1290	69	723	140	S7
Yolk–shell	0.01–3.0	300 mA g^{-1}	1177	76	848	80	S8
Hollow microcube	0.01–3.0	100 mA g^{-1}	1522	72.4	457	100	S9
Graphene-constructed hollow sphere	0.01–3.0	100 mA g^{-1}	1353	82.1	950	50	S10
Hollow cubic	0.005–3.0	0.1 C	1603	60.5	576	60	S11
Multi-shelled hollow sphere	0.05–3.0	400 mA g^{-1}	1360	72	861	50	S12
Porous multi-shelled hollow sphere	0.01–3.0	100 mA g^{-1}	1313	78.4	869.9	300	S13

References

S1. Cho, J. S., Hong, Y. J., Lee, J. H. & Kang, Y. C. Design and synthesis of micron-sized spherical aggregates composed of hollow Fe_2O_3 nanospheres for use in lithium-ion batteries. *Nanoscale* **7**, 8361-8367 (2015).

S2. Wang, B., Chen, J. S., Wu, H. B., Wang, Z. & Lou, X. W. Quasiemulsion-templated formation of α-Fe_2O_3 hollow spheres with enhanced lithium storage properties. *J. Am. Chem. Soc.* **133**, 17146-17148 (2011).

S3. Zhou, J. *et al.* Carbon-encapsulated metal oxide hollow nanoparticles and metal oxide hollow nanoparticles: a general synthesis strategy and its application to lithium-ion batteries. *Chem. Mater.* **21**, 2935-2940 (2009).

S4. Zhu, J. *et al.* Hierarchical hollow spheres composed of ultrathin Fe_2O_3 nanosheets for lithium storage and photocatalytic water oxidation. *Energy Environ. Sci.* **6**, 987-993 (2013).

S5. Zhang, L., Wu, H. B., Madhavi, S., Hng, H. H. & Lou, X. W. Formation of Fe_2O_3 microboxes with hierarchical shell structures from metal–organic frameworks and their lithium storage properties. *J. Am. Chem. Soc.* **134**, 17388-17391 (2012).

S6. Sasidharan, M., Gunawardhana, N., Yoshio, M. & Nakashima, K. α-Fe_2O_3 and Fe_3O_4 hollow nanospheres as high-capacity anode materials for rechargeable Li-ion batteries. *Ionics* **19**, 25-31 (2013).

S7. Du, Z., Zhang, S., Zhao, J., Wu, X. & Lin, R. Synthesis and characterization of hollow α-Fe_2O_3 spheres with carbon coating for Li-ion Battery. *J. Nanosci. Nanotechnol.* **13**, 3602-3605 (2013).

S8. Son, M. Y., Hong, Y. J., Lee, J. K. & Kang, Y. C. One-pot synthesis of Fe_2O_3 yolk–shell particles with two, three, and four shells for application as an anode material in lithium-ion batteries. *Nanoscale* **5**, 11592-11597 (2013).

S9. Xiao, H. *et al.* Template-free synthesis of hollow α-Fe_2O_3 microcubes for advanced lithium-ion batteries. *J. Mater. Chem.* A **1**, 2307-2312 (2013).

S10. Chen, Y. *et al.* Self-assembled graphene-constructed hollow Fe_2O_3 spheres with controllable size for high lithium storage. *RSC Adv.* **5**, 21740-21744 (2015).

S11. Wu, C. *et al.* Synthesis and the comparative lithium storage properties of hematite: hollow structures vs. carbon composites. *RSC Adv.* **5**, 21405-21414 (2015).

S12. Padashbarmchi, Z. *et al.* A systematic study on the synthesis of α-Fe_2O_3 multi-shelled hollow spheres. *RSC Adv.* **5**, 10304-10309 (2015).

S13. Wu, Z. G. *et al.* L-histidine-assisted template-free hydrothermal synthesis of α-Fe_2O_3 porous multi-shelled hollow spheres with enhanced lithium storage properties. *J. Mater. Chem.* A **2**, 12361-12367 (2014).
